# Marking the markers: evaluating the potential of professional development through collaborative marking circles

**DOI:** 10.1042/ETLS20253033

**Published:** 2026-02-26

**Authors:** Stephen Rutherford, Connie Pritchard, William Kay, Larissa Nelson, Hannah Shaw, Nigel Francis

**Affiliations:** 1 School of Biosciences, Cardiff University, Cardiff, CF10 3AX, U.K.

**Keywords:** assessment, collaborative marking, higher education, inter-rater reliability, marking rubrics, professional development, reflective practice

## Abstract

This case study details a three-day ‘marking circle’ activity where six academics, who co-author this case study, with varied lengths of experience collaboratively marked 25 undergraduate bioscience essays. Observations from this activity, discussed in a focus group, highlighted a significant tension: while the process was too time-consuming to be a sustainable method for routine marking, it was an extremely valuable and enjoyable professional development exercise. The activity revealed substantial inconsistencies between markers, with mark variations often exceeding 20%. In a focus group discussion between the markers, these discrepancies were attributed to several elements: procedural, structural, personal and experiential factors that led to different decisions between the markers when assigning numerical grades. This case study proposes a streamlined, two-stage model to harness the activity’s developmental benefits in a more time-efficient manner: first, using a small group to pilot and refine a rubric, and second, using the refined rubric in a wider-scale professional development context.

## Introduction

The reliability of assessment is fundamental to the integrity of student performance in higher education (HE). Confidence in the reliability of evaluations of students’ capabilities directly affects the validity of degree outcomes. A major concern for reliability of marking is how to ensure consistency between markers (inter-rater reliability) when marking extended written assignments [1,2]. The implicit subjectivity of marking highly variable and nuanced work is challenging. Approaches to enhance inter-rater reliability often include use of detailed marking rubrics, designed to norm-reference the judgements of assessors and standardise the application of criteria [3]. The underpinning assumption of the use of rubrics is that a shared framework can mitigate variation between personalised interpretations of quality [4].

Despite the widespread use of rubrics, there is evidence that variation in grading practices in HE persists [5,6]. Rubric efficacy can be limited by ambiguous descriptive language and the influence of overriding assessor expectations and preconceptions, including individual biases, as well as subject expertise [2,3,7]. Therefore, using a shared assessment instrument (such as a rubric) in isolation may be insufficient to guarantee consistency between markers. A more process-oriented mechanism is also required to align marker expectations [8].

Collaborative, peer and dialogic assessment practices have been explored for developing shared understandings of academic standards between assessment markers. Such activities provide opportunities for peer discussion and vocalisation of the justifications for decisions [9,10]. Collaborative processes can enhance marking consistency and also serve as valuable continuing professional development (CPD) for those involved [11,12].

This paper presents a case study of a ‘marking circle’, a collaborative, peer-based marking activity. The case study presents observations of the activity, with particular focus on the potential implications for CPD. The case study aims to highlight impacts on both marking validity and professional awareness of markers [4]. The case study evaluates the potential of the marking circle as a professional development tool for markers and suggests ways in which the activity could be streamlined for wider adoption.

## Methodology

### Essay pool

Twenty-five undergraduate bioscience essays (approx. 1500 words) were selected, with the authors’ permission, from a single Year 1 summative assignment undertaken in spring 2024. The assignment included a choice of 21 essay titles for students; essays chosen for this activity were selected to represent a range of mark outcomes and essay titles. 9 essay titles were selected (3 essays each for 8 titles, plus a single essay for the ninth). All essays had been marked previously by a pool of >100 academic staff. The grades and feedback of the Original Marker (‘OM’) were noted, but not shared with the markers during this activity.

### The marking exercise

The marking circle comprised six colleagues, the co-authors of this paper. The markers were all academic staff, with varying lengths of experience marking undergraduate essays, ranging from 0 to 27 years (Table 1). The activity took place face-to-face over three days, with approximately six hours dedicated to the task each day. None of the markers were experts for all essay subjects, but some did have subject expertise in a range of essay titles. The essays for which individual markers had subject expertise are noted in Table 1. As Level 4 (Year 1 undergraduate) subjects, however, the essays were sufficiently accessible to all of the markers (all Bioscience educators) as to make the marking of all essays valid.

**Table 1:** Marker summary

Marker	Years of experience	Essay numbers within discipline area
1	0	7, 24, 25
2	5	3, 4, 5, 13, 14, 20, 21, 22, 23,
3	13	8, 9, 10, 15, 16, 17
4	14	6, 18, 19
5	17	8, 9, 10, 15, 16, 17
6	27	4, 5, 6, 7, 13, 14, 20, 21, 22, 23, 24, 25

**Figure 1:**
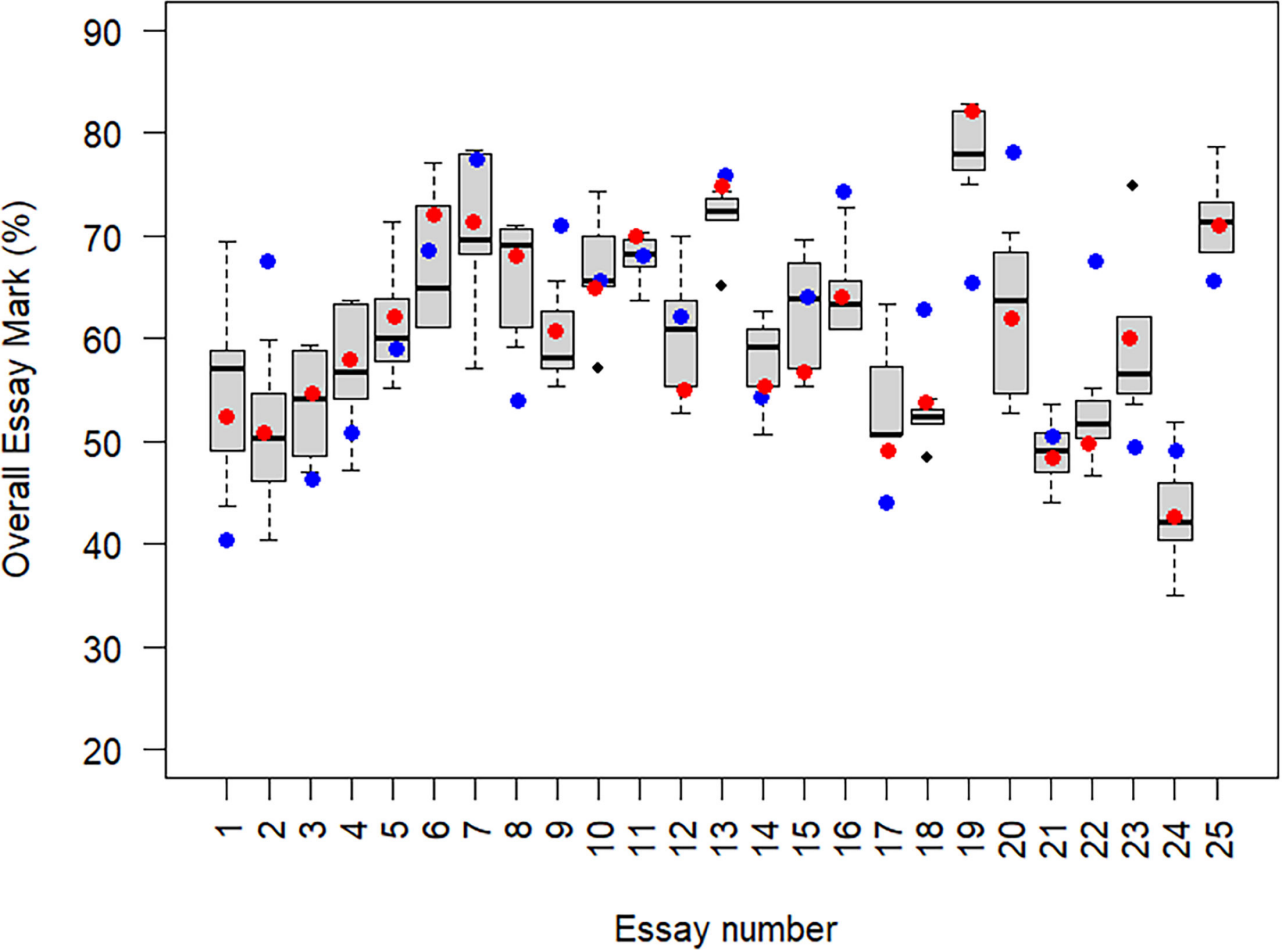
Spread of marks across all essays.

The years of experience grading work in higher education for the six markers. Marker 1 had not marked undergraduate essays before this exercise. The numbers of the essays in Figure 1 which fall under the disciplinary expertise of each marker is indicted. Essays 1, 2, 11 and 12 did not have subject experts within the marking team.

Marking used a rubric (see Table 2) with four marking criteria: two criteria focused on content/comprehension and two criteria focused on presentation and use of references. Marks were awarded across a 100% scale, divided into a 20-point categorical marking scale, spread between seven written descriptors (most descriptors were a 10% range with 2, 5 and 8 points within the range). When marking the essays, each criterion was graded separately. The four criteria were differentially weighted (see Table 2 legend) to produce an overall mark.

**Table 2:** The scoring rubric

		% mark	0, 15, 25, 35	42, 45, 48	52, 55, 58	62, 65, 68	72, 75, 78	85	95, 100
			Fail	Pass	Satisfactory	Good	Excellent	Exceptional	
**Criteria**	1	Relevance and coverage of content, knowledge and understanding of core principles and theories.	Descriptor 1.1	Descriptor 1.2	Descriptor 1.3	Descriptor 1.4	Descriptor 1.5	Descriptor 1.6	Descriptor 1.7
	2	Organisation and communication of evidence and ideas appropriate for the audience	Descriptor 2.1	Descriptor 2.2	Descriptor 2.3	Descriptor 2.4	Descriptor 2.5	Descriptor 2.6	Descriptor 2.7
	3	Presentation skills/ use of style, colour, font headings, balance of text and images	Descriptor 3.1	Descriptor 3.2	Descriptor 3.3	Descriptor 3.4	Descriptor 3.5	Descriptor 3.6	Descriptor 3.7
	4	Use of supporting evidence/ literature	Descriptor 4.1	Descriptor 4.2	Descriptor 4.3	Descriptor 4.4	Descriptor 4.5	Descriptor 4.6	Descriptor 4.7

Each of the four criteria was scored across seven mark boundaries. Each mark boundary had a single explanatory descriptor for each criterion. Within each boundary were typically three mark points (e.g. 42%, 45% and 48%, 52%, 55% and 58%), with the ‘fail’ and ‘exceptional’ ranges having different mark points. The proportionate weights of the four criteria (1–4) were in the ratio of 3:1:1:2 (they represented 35% of the mark in the original assessment).

Markers assigned marks to each criterion independently and were not shown the marks or feedback from the original marker of the essays.

For each essay, markers read the work simultaneously and privately assigned a numerical mark for each criterion using the rubric. When complete, taking each criterion sequentially, individuals revealed and justified their mark before the group agreed on a consensus mark for that criterion and moved to the next. To ensure equal participation, the first speaker in the discussion rotated for each essay.

### Discussion of the process

The six markers met online via MS Teams to discuss their experiences of the marking process in a focus group. The discussion lasted 76 minutes, with Marker 2 leaving after 50 min. Discussion began with an open question of ‘Can you tell us how you found the marking process?’, and the discussion evolved iteratively from that point. The discussion was recorded with the permission of all markers and converted into a written transcript for analysis.

### Analysis of the focus group data

Qualitative analysis was undertaken by one team member using ‘Constructivist Grounded Theory’ (Charmaz, 2011) as a methodology. This approach was chosen above a thematic analysis approach in order to avoid overlaying any prior assumptions on the outcomes of the analysis. The transcripts were coded, the codes clustered into categories, and then into themes for further consideration. Those categories relating to the insights from this exercise for assessment-related CPD are discussed below.

## Observations from the activity

A series of observations were made during the course of the marking circle, both on the value of the activity itself, and the potential impact on the markers. Figure 1 shows the degree of variation of composite essay mark (the weighted average of the four criteria) allocated by the 6 markers, compared with the original marker’s mark and the agreed group mark. There was substantial variation between markers, often by 10% or more. The nuances of the mark variations are not discussed in detail in this analysis and evaluated elsewhere (Kay et al., in preparation). However, the variation of the marks awarded was sufficient to warrant reflection on the marking processes that might lead to such variation. This case study aims to highlight observations related to the markers’ perceptions of the value of this collaborative marking exercise as a potentially insightful CPD activity.

The spread of marks for the 25 essays is represented as box-plots. The essays are ordered in sequence of blue dot = OM mark. Red dot = Agreed group mark. Statistical analysis and figure produced using R Studio.

### The activity was time-consuming

The activity took three days to complete 25 essays, requiring approx. 45 min per essay. This time commitment was recognised as substantial, and throughput was too low for this to be a sustainable marking activity. Even as a CPD exercise, markers noted the high time investment was problematic.

### The activity was enjoyable

Despite the amount of time taken on the process, all of the markers observed that the process itself had not only been useful, but had actually been enjoyable! The markers all noted how potentially valuable this would be for colleagues to undertake as a CPD activity.

I thoroughly enjoyed it, and I think it’s such an eye-opening experience for people who are interested and committed to this kind of area.

Marker 4

I also really enjoyed it. It was something I’ve been wanting to do for a really long time and it’s something I’ve actually suggested to (School Assessment Lead) on a number of occasions

Marker 5

Although discussions were sometimes intense, the positive value to understanding themselves and their peers was deemed worth the investment. The social interaction, frank discussions, mutual recognition of marking challenges and resulting camaraderie created a positive atmosphere. This outcome was achieved through mutual respect, which is critical in an exercise that could potentially leave markers (particularly those who are less experienced) feeling vulnerable. Markers 1 and 2, the more junior colleagues, noted how the exercise had not only helped develop their marking, but had been supportive and not intimidating.

For a very early career educator, for me that’s been really beneficial … it’s highlighted my own areas where I need to pay more attention to …, that’s been a great learning experience for me.

Marker 1

I also really enjoyed the experience. I had brief, you know, … only brief moments of imposter syndrome.

Marker 2

### Impact on peer-bonding and understanding

The activity significantly improved markers’ understanding and respect for their colleagues’ perspectives and individual differences. Working closely, discussing differing opinions respectfully and listening to each other’s rationales enhanced professional integration. Every member, regardless of their levels of experience, reported that they had learned and developed, gaining better insight into their colleagues’ ways of thinking and working.

### Highlighting issues of consistency between markers

The marking circle revealed substantial differences between individual markers (Figure 1). There was never a clear group consensus of a single mark for a criterion. Criterion marks sometimes varied by >20% between markers, highlighting considerable inconsistency between interpretations of quality. Discussion in the focus group identified a range of factors that could potentially impact the markers’ perceptions of quality. Discussion of these variable factors in the focus group highlighted subconscious marker biases and expectations. The markers all identified that these biases existed, and equally that they, for the most part, had not recognised them before.

The amount of bias that we all had with regards to specific elements of the criteria I thought was absolutely fascinating, and how that can change with different essays.

Marker 5

It really helped me identify where my biases are, and I haven’t really stopped and considered that before because they’re just set, they’re innate and because we don’t mark in teams, we’ve never really discussed those.

Marker 4

**Figure 2:**
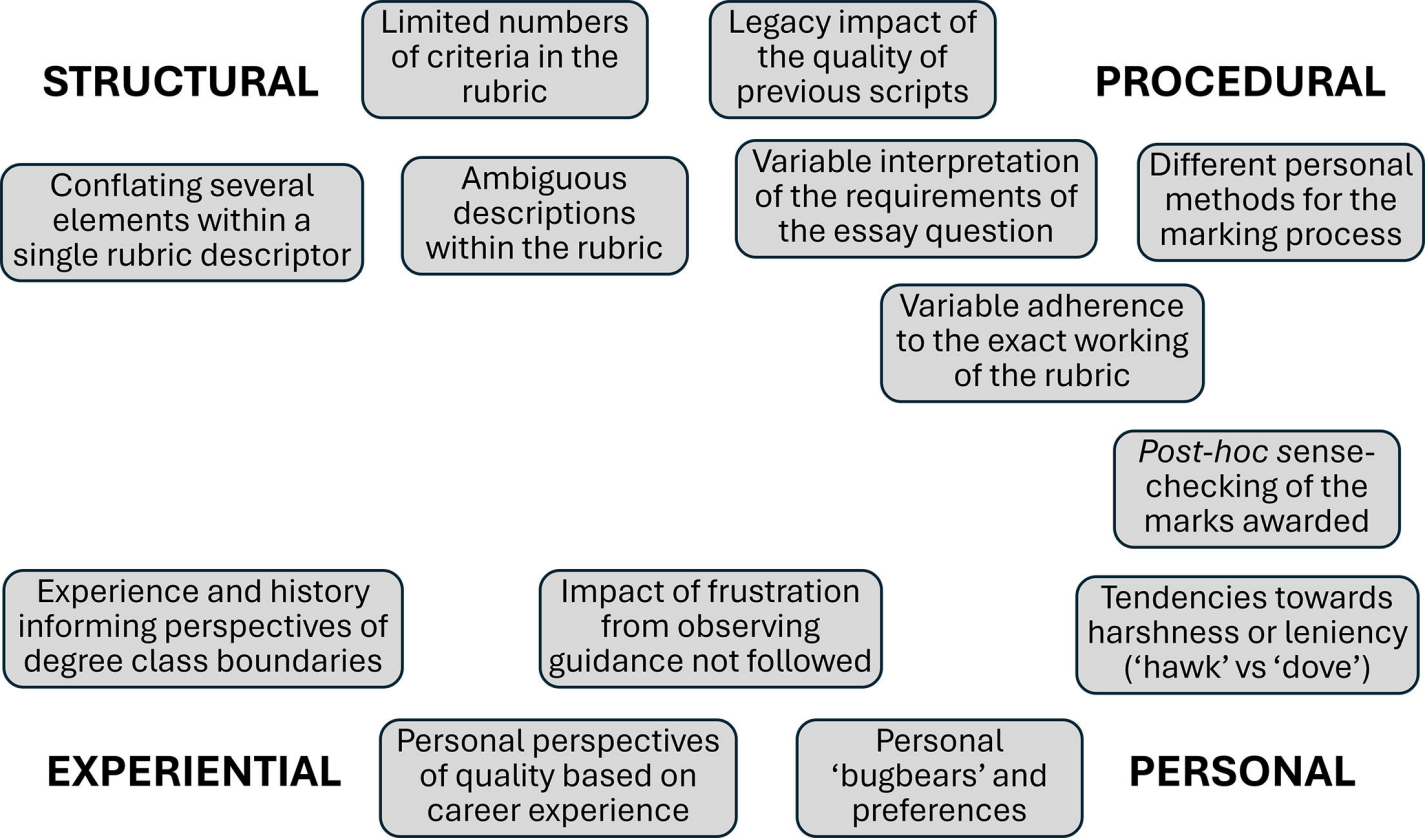
Factors affecting inter-rater variability.

The range of factors identified by the markers are summarised in Figure 2. These factors highlighted in the discussion fell between four AREAS: *structural* (issues relating to ambiguity of the rubric descriptors, or bluntness of the rubric); *procedural* (variations in how individual markers approached the marking, interpreted the rubric descriptors or interpreted the importance of the requirements of the different essay questions (e.g. ‘describe’ vs ‘discuss’)); *personal* (innate biases, tendencies towards harshness or leniency); *experiential* (personal perspectives of quality, developed over the course of the marker’s career).

Various factors (grey boxes) are positioned relative to their alignment with four factors: Structural, procedural, personal and experiential. Positioning of the boxes relative to the four factos is indicative only, not quantitative.

The discussion surfaced that these factors influencing inter-rater variation were themselves the result of two intersecting elements: perceptions of quality, and the process of converting that quality judgement into a numerical mark. The process of assigning the numerical mark related more to the procedural and structural factors, while perceptions of quality related more to the experiential and personal factors.

I found it reassuring almost that even though we have our differences in opinions, a lot of the times … we were in agreement with the feedback that we were giving. The differences were in the numbers that we placed to that. But I felt it quite reassuring that we were all kind of thinking the same thing, even if then we replaced it with a number.

Marker 3

These factors were all recognised as important elements that would be important to surface within a CPD exercise, for markers to reflect upon. Surfacing biases and expectations empowered markers to recognise their existence, and through this potentially develop strategies to mitigate their impact on their decision-making. Recognition of the true extent of the variety of perspectives also emerged. Observing the substantive differences in marks awarded using the same rubric on the same essay underscored the extent of subjectivity within their marking approaches to the markers.

### Time taken versus learning gain

Although the exercise was time-consuming, all markers agreed the learning gains were considerable. Insights could be personal realisations or reflections, observations of how others work, adopting new approaches that others found useful, or identifying potentially problematic approaches that needed to be abandoned. But they recognised that this time commitment was likely to be a barrier.

I think it has been really, really time-consuming, and I don’t think a lot of staff would be willing to give up three days to mark 25 essays. So, I think there needs to be a way that we can emphasise the importance and the value of this process, but also streamline it a bit. So that it’s something that can be done in an hour, or two hours, and how do we get people to buy into this as a concept and highlight that it adds value to the marking teams.

Marker 4

The most time-consuming aspects, however, were identified to be a consequence of structural and procedural factors, and the ambiguity within the rubric descriptors. Marker 6 suggested that refining this could lead to the exercise being more streamlined.

I think as well, you can get around an awful lot of that time, because I think the reason it took us half an hour (40 minutes sometimes) to (assign a mark) was because we were trying to interpret the mark criteria. So, I think if you had marketing criteria that are fit for purpose, we would have had much shorter discussion times.

Marker 6

Despite the time commitment, it was felt that the activity surfaced several issues that could inform improvements to the assessment, and broader marking policies, and prompted discussions of specific challenges like Generative AI, and issues of co-ordination between advice given to students and guidance for markers.

## Discussion

The potential benefits of this as an exercise for CPD were substantial. The marking circle provided a wide range of insights, personal and policy-related, which would benefit both the markers and course management overall. The benefit of surfacing marking processes was clear and led to valuable discussions of personal influences, procedures, expectations and values. The activity also revealed fundamental flaws in the marking process as it currently stood. The marking circle activity was recognised as beneficial by all markers, as a valuable use of their time and effort, and a potentially valuable CPD activity.

The two elements of determining quality and assigning the mark have different implications for professional development. The structural and procedural factors influencing assignment of the numerical mark could be easily addressed by refining the rubric and better-aligning the wording of essay questions to reduce ambiguity and potential for subjective misinterpretations. Judgements of quality, however, are potentially long-term challenges to address, and require surfacing values, biases and influences that the markers might not previously have considered. This observation infers that there are two distinct goals for any CPD activity – refining the process and aligning expectations of quality.

### Suggestions for practice

**Figure 3:**
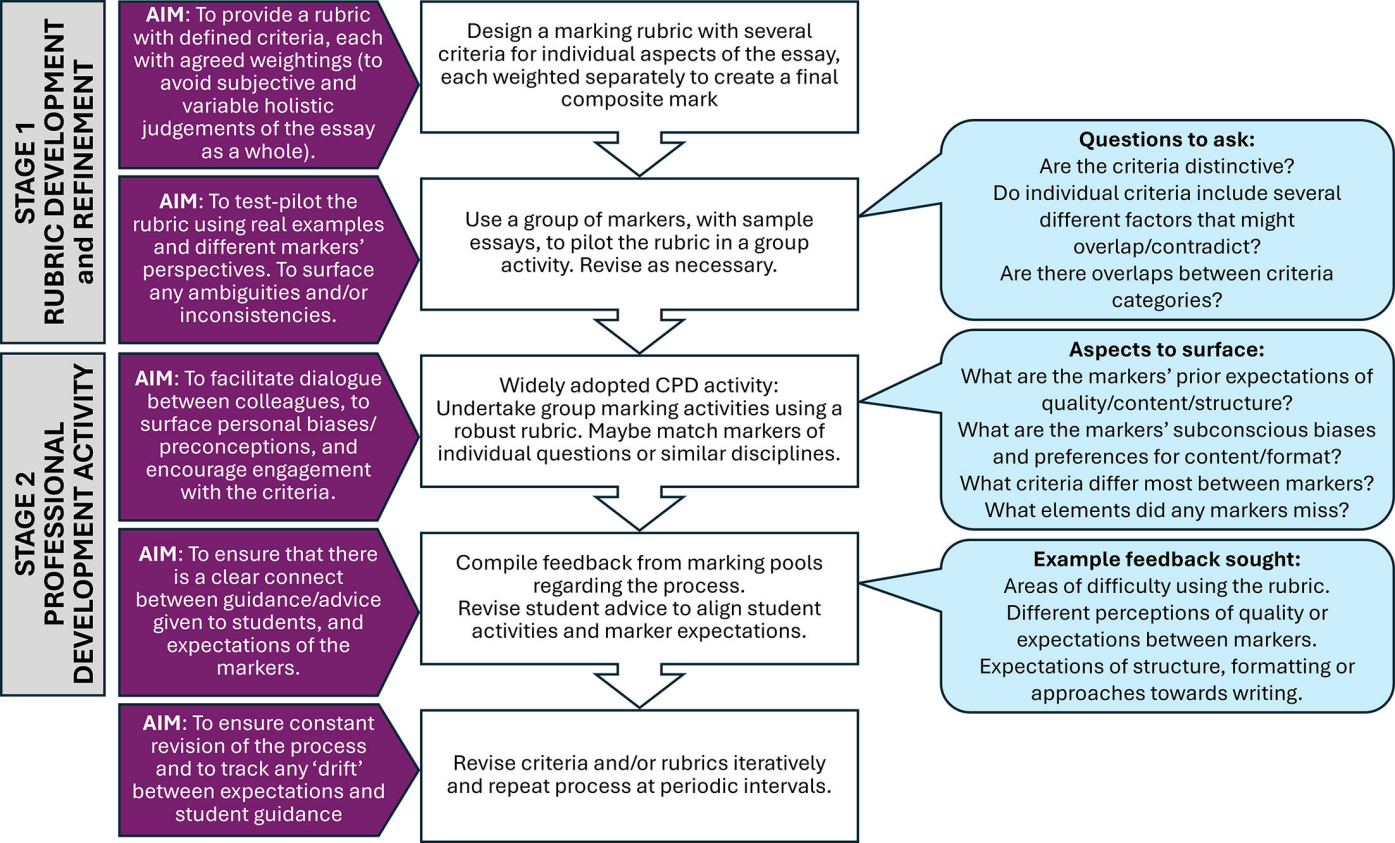
Suggested approach for replicating this as a CPD activity.

Even though this group marking activity appears to be valuable, the three-day commitment is a significant barrier to wider adoption, as academics are typically time-poor. Streamlining the process is therefore necessary to maximise benefit with minimal time investment. Key to this streamlining may be the refining of the rubric. The majority of longer discussions focused around defining a specific mark, rather than the overall assessment of the essay’s quality. Therefore, making the process of assigning the numerical mark more robust may be the most impactful factor. A process of refining the rubric to ensure that it is fit for purpose, by using it actively within a peer-based setting, would potentially enable the marking process to require less discussion. An efficient process may therefore be a two-stage activity (summarised in Figure 3):

#### Stage 1: rubric piloting

The process is divided into two stages, each involving different colleagues. Stage 1: A small group of colleagues refine the rubric. Stage 2: A wider community of educators adopts this as CPD. The purple arrows highlight the aims of each stage, the white boxes show each activity, and the blue speech bubbles identify the key questions to be asked, or insights to be looked for.

A small group of colleagues with varied career experience would use the draft rubric to mark a sample of previous student work. The key aim is to identify and resolve flaws, ambiguities and inconsistencies within the rubric and the marking process before it is used for live assessment. Less-experienced colleagues are valuable in this process, as they have fewer ingrained presumptions of quality and may therefore be more objective in identifying flaws in rubrics and procedures.

#### Stage 2: professional development

The revised, fit-for-purpose rubric would then be used in a wider-scale CPD exercise with a smaller sample of student work (e.g. 4–6 essays per group). The aim here would be to calibrate standards by identifying inconsistencies in perception, surfacing personal biases and discussing the application of the criteria. This should dually enhance marking reliability while providing valuable professional insights but require only a few hours commitment.

### Implications for use in CPD

The experience of this exercise corroborates previous research identifying the benefits of collaborative marking [10,12]. Significant mark variations were observed, despite the use of a detailed rubric. This emphasises inter-rater variability in the assessment of undergraduate written work [2]. A primary cause of variation appeared to be ambiguity within the rubric, reflecting what Sadler [1] describes as ‘inherent indeterminacy’ in the application of grading criteria.

The implications for CPD of individual academics are considerable. The marking circle functioned as a powerful mechanism for reflective practice [13], enabling markers to develop metacognitive awareness of their personal evaluative frameworks [2]. The surfacing of previously unrecognised influences on perceptions of quality provided a conceptual tool for academics to review their own reflexive marking tendencies, a known challenge in HE assessment [14]. The activity potentially supports long-term development of a community of practice in assessment, enhancing mutual professional respect and understanding among colleagues [9].

An intensive, peer-based marking activity such as this exercise could be an effective intervention. However, a major consideration is the substantial resource investment required, making the marking circle unsustainable as a routine marking procedure. Consequently, a primary contribution of this paper is the proposal of a resource-efficient, two-stage protocol, separating the critical functions of rubric refinement and professional development. Applying this activity as a CPD exercise can empower markers to recognise their own preconceptions, but also how widely those preconceptions may differ from their colleagues. It is hoped that this revelation might lead to improved rubrics, closer rubric adherence, better guidance to students and higher validity of awarded marks.

## Open Access

Open access for this article was enabled by the participation of Cardiff University in an all-inclusive Read & Publish agreement with Portland Press and the Biochemical Society under an agreement with JISC.

## Abbreviations

CPD: continuing professional development
HE: higher education
OM: Original Marker
